# Thymic Stromal Lymphopoietin in Cutaneous Immune-Mediated Diseases

**DOI:** 10.3389/fimmu.2021.698522

**Published:** 2021-06-24

**Authors:** Si-Hang Wang, Ya-Gang Zuo

**Affiliations:** Department of Dermatology, National Clinical Research Center for Dermatologic and Immunologic Diseases, Translational Medicine Center, Peking Union Medical College Hospital, Chinese Academy of Medical Sciences and Peking Union Medical College, Beijing, China

**Keywords:** thymic stromal lymphopoietin, inflammation, allergy, autoimmunity, skin diseases

## Abstract

Thymic stromal lymphopoietin (TSLP) was initially demonstrated to be critical in regulating inflammatory responses among various allergic disorders (such as atopic dermatitis, food allergy, and asthma). Although two isoforms (short form and long form) of TSLP have been demonstrated in human tissues, the long form of TSLP (lfTSLP) is strongly implicated in the pathogenesis of allergies and cutaneous immune-mediated diseases. The immunomodulatory activity of lfTSLP varies widely, driving T helper (Th) cells polarizing Th2 and Th17 immune responses and inducing itch. Moreover, lfTSLP is closely associated with skin fibrosis, epidermal hyperplasia, angiogenesis, and homeostatic tolerogenic regulations. This review highlights significant progress from experimental and clinical studies on lfTSLP in cutaneous immune-mediated diseases (atopic dermatitis, psoriasis, bullous pemphigoid, systemic sclerosis, chronic spontaneous urticaria, Behçet’s disease, vitiligo, rosacea, systemic lupus erythematosus, and alopecia areata). We also offer original insights into the pleiotropic properties of the cytokine TSLP in various pathophysiological conditions, with significant clinical implications of TSLP-targeted therapies for immune-mediated skin diseases in the future.

## Introduction

Thymic stromal lymphopoietin (TSLP), a distant paralog of interleukin (IL)-7, is a pleiotropic molecule within the IL-2 family (1, 2). Murine TSLP was initially detected in a conditioned medium from the supernatant of a thymic stromal cell line, sustaining the growth of B cells and modulating thymocytes proliferation *in vitro* (3, 4). When added to long-term bone marrow or fetal liver cultures, TSLP stimulated the B220+/IgM+ B cells expressing k light chains on their surfaces (4). Two isoforms for TSLP [long-form TSLP (lfTSLP) and short-form TSLP (sfTSLP)] were first determined by Harada et al. in human bronchial epithelial cells (5). The functional and high‐affinity receptor of lfTSLP isoform includes both the TSLP receptor (TSLPR) subunit (binding to TSLP with low affinity) and the IL-7 receptor α-chain (6, 7). Human homology of TSLP was initially identified by *in silico* methods in 2001 (8, 9). The human TSLP gene is mapped to chromosome 5q22.1, near the atopic molecular cytokine cluster (8, 9). Its protein has two isoforms consisting of 159 amino acids in length, and the other, 63 amino acids (5, 9). Murine TSLP is a 140-amino acid protein located on murine chromosome 18 (1, 5, 8, 9). Human and murine TSLP/TSLPR signaling pathways share similar functional characteristics despite relatively low genetic homology, indicating the TSLP/TSLPR axis is conserved among humans and mice (10, 11). During the initiation of inflammation, the principal sources of TSLP are keratinocytes, epithelial cells, fibroblasts, mast cells, macrophages, and dendritic cells (DCs) (10, 12, 13). The functional TSLPR is expressed by DCs, innate lymphoid cells (ILCs), mast cells, B cells, T cells, eosinophils, monocytes, and several non-hematopoietic cell populations such as epithelial cells (10, 12–17).

Over the past two decades, experimental and clinical studies have demonstrated TSLP to be a potent initiator of type 2 allergic inflammatory responses in humans and mice (11). Furthermore, multiple reviews have described the role of TSLP in allergic disorders [atopic dermatitis (AD), airway allergy, and food-hypersensitivity reaction], autoimmune diseases, infections, and cancers (10, 11). Moreover, TSLP has also been linked to intestinal protection and homeostatic tolerogenic regulations (18). Epithelial cell-intrinsic TSLP has a significant impact upon tolerogenic DCs generation that promotes the regulatory T cell differentiation in the intestine and thymus (19–21). This review will focus on emerging messages on lfTSLP-related allergic disorders and immune-mediated skin diseases, adding new insights into the pleiotropic properties of lfTSLP in various pathophysiological conditions.

## A Dichotomy Between Two Isoforms of TSLP

Emerging data have provided more substantial evidence of a dichotomy between the variants of TSLP in humans: lfTSLP and sfTSLP (5). SfTSLP is short of the N-terminal sequence of lfTSLP but shares the identical domain at the C-terminal (5). This group further revealed that the TSLP promoter polymorphisms of the two SNPs Rs2289276 and Rs2289278 increase transcription factor activating protein-1 binding and subsequent lfTSLP production. In that way, the promoter SNPs correlate with the susceptibility of asthma in children and adults (22).

The mRNA derived from an internal promoter in intron 2 of TSLP has been predicted to encode the sfTSLP, consisting of 63 amino acids (5). Interestingly, the mRNA was constitutively expressed in keratinocytes, epithelial cells, and lung fibroblasts (5). This expression remains unchanged even after challenging with lipopolysaccharide capable of inducing lfTSLP transcription (5, 23–26). However, the expression of sfTSLP at the protein level and its physiological role remain unknown. Human sfTSLP mRNA is the predominant isoform of TSLP in keratinocytes and salivary glands (26). Several studies demonstrated sfTSLP is not regulated similarly to lfTSLP and may exert antibacterial and anti-inflammatory effects (26–30). Although these experiments have suggested sfTSLP is implicated in homeostatic conditions and inflammations, further investigations are needed to determine whether it is expressed as a functional protein *in vivo* and the mechanism of regulation in the signaling pathways involved in sfTSLP (for example, the contribution of vitamin D3 and nuclear receptor peroxisome proliferator activated receptor-γ) (25, 27). Next, this review will exclusively describe lfTSLP and its participation in allergies and cutaneous immunities

## TSLP in Allergy

**Figure 1:**
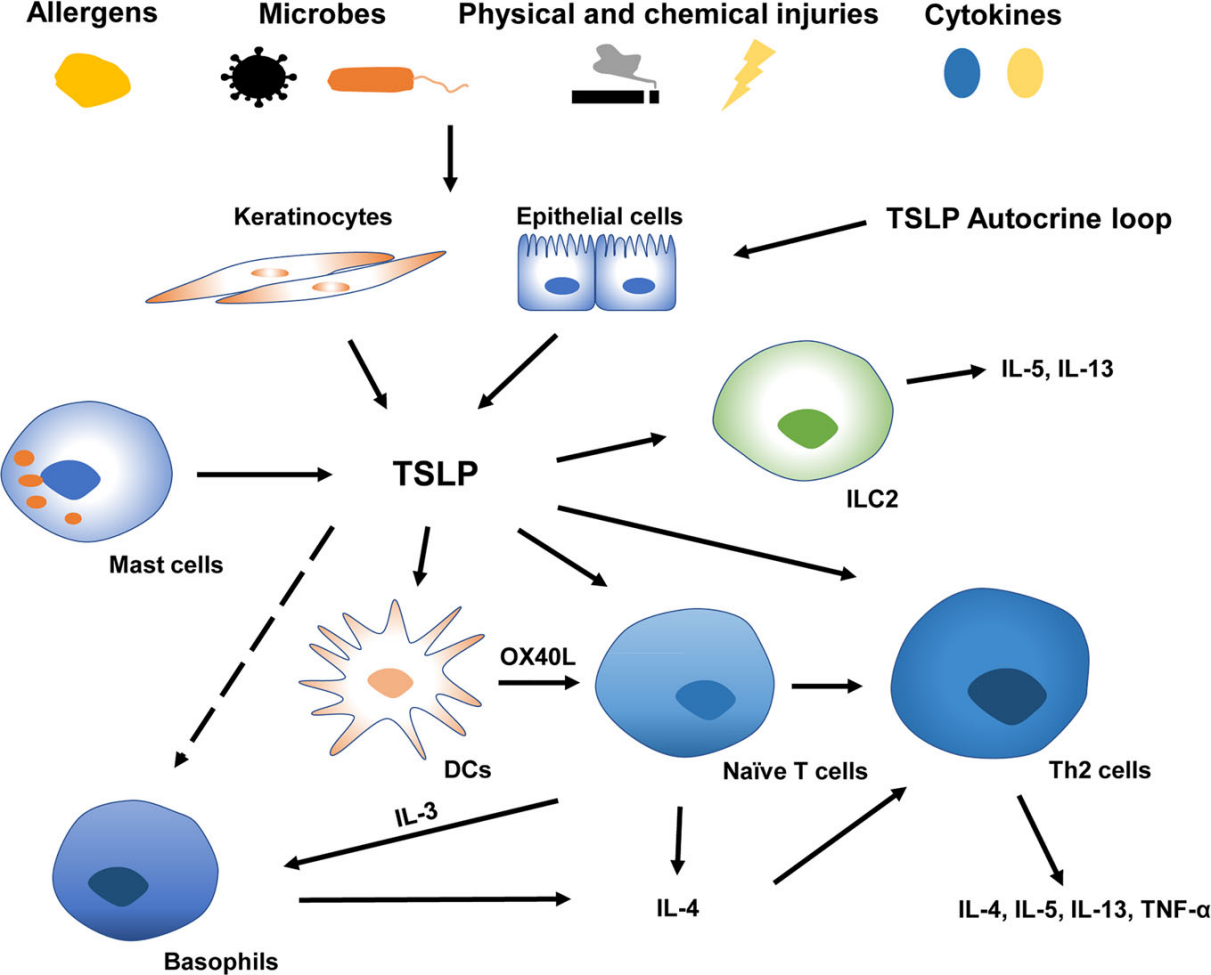
TSLP in type 2 allergic inflammatory responses at barrier surfaces. A variety of endogenous or environmental factors may contribute to the production and release of TSLP from barrier surfaces. Keratinocytes and epithelial cells derived TSLP activates DCs, ILC2, naïve CD4+T cells, and Th2 cells (Whether TSLP directly affects basophils remains controversial). TSLP-triggered DC induces naïve T cells to release IL-3 *via* OX40L, which is capable of recruiting basophils polarizing Th2 inflammation. TSLP, thymic stromal lymphopoietin; IL, interleukin; Th2 cells, type 2 T helper cells; OX40L, OX40 ligand; DCs, Dendritic cells; ILC2, type2 innate lymphoid cells.

TSLP’s roles in type 2 allergic responses have been extensively studied (**Figure 1**). Type 2 immunity, characterized by the abundance of CD4+T helper 2 (Th2) cells, eosinophils, mast cells, basophils, and group 2 ILCs, prevents the host from extracellular parasitic insult but causes chronic allergy (31). During the initiation of Th2 responses, epithelial exposure to stimuli such as proteolytic allergens, bacteria, parasites, and chemicals triggers a variety of protease activating receptors and pattern recognition receptors on barrier epithelial cells, inducing the production of TSLP, IL-25, and IL-33. Furthermore, the generation of endogenous danger-associated molecular patterns can amplify the release of pro-Th2 cytokines by epithelial cells (31). DCs triggered by TSLP induce naive T cells to differentiate into Th2 cells through co-stimulatory molecules CD86 and OX40L (32). This DC activation may further enhance Th2 allergic inflammation *via* macrophage-derived and thymus and activation-regulated chemokine production (12). Subsequently, these cytokines/chemokines result in the production of Th2 molecules IL-4, IL-5, IL-13, and tumor necrosis factor (TNF)-α by CD4+T cells and reduction of IL-10 and IFN-γ (11, 12, 32). Additional research showed that TSLP induced the rapid Th2 cell maturation *via* the TSLPR expressed on naïve CD4+T cells, indicating a possible earlier activation of T cells triggered by TSLP (33, 34). Moreover, TSLP can modulate the proliferation of Th2 cell linage through high-level TSLPR on rodent effector T cells (35). It has also been implicated in innate immunity, promoting the release of type 2 cytokines from mast cells (14) and priming type 2 ILCs to express IL-5 and IL-13 in an IL-25 and IL-33-independent manner (36). Additionally, TSLP may contribute to the amplification of macrophage polarization (37).

TSLP’s role in activating basophils has been intensively studied in the past decade, and the role of TSLP in triggering basophils in Th2 differentiation remains highly controversial (16, 38, 39). Basophils can initiate and enlarge inflammation by secreting IL-4 and are linked with Th2 immune responses (40). A previous study using a vitamin D3 analog MC903 (calcipotriol) -induced mouse model revealed that DCs activated by TSLP prime naïve T cells to secret IL-3 *via* OX40L, and IL-3 could recruit basophils resulting in Th2 priming (41). The cascade characterized by orchestrated “DC-T-basophil-T” through the OX40L-IL-3 axis precedes the production of IL-4 by T cells so that basophils could provide an earlier source of IL-4 to prime type 2 immune responses (41). Other studies further demonstrated that the “TSLP-basophil-IL-4” signaling is crucial in Th2 polarization (42, 43). TSLP and basophils are closely associated with the development of intestinal food allergy in response to epicutaneous sensitization with food antigen (44, 45). Critically, TSLP-induced basophil is related to the evolution of allergy from the skin to the remote gastrointestinal mucous membrane *via* IL-4 (42). This sequence was delineated as the “atopic march” characterized by a sequential disease progression from AD to food allergy and asthma (42, 46).

Nevertheless, Whether TSLP directly activates basophils remains disputed (39). Several studies have illustrated heterogeneity within basophil cell lineages (TSLP-elicited and IL-3-elicited basophils). They claimed that TSLP affected bone-marrow cells to enhance basophil hematopoiesis independently of IL-3, suggesting that basophil activation was driven at least in part by TSLP (16, 38). Several clinical experiments showed the upregulation of TSLPR in patients with allergic rhinitis and asthma after allergen stimulation. However, TSLPR expression on basophils is minimally associated with asthma severity, indicating a limited role for basophils in TSLP-induced inflammation (17, 38, 47). Based on these findings, basophils are involved in the polarization, expansion, and progression of Th2 polarization and appear to be critical in the pathogenesis of AD, allergic asthma and rhinitis, and eosinophilic chronic rhinosinusitis (48). However, the mechanism of the activation of basophils by TSLP under these abnormal conditions warrants additional investigations. Moreover, tactics targeting the “basophil-IL-4” pathway may prevent the evolution of type 2 allergic responses from the skin to internal organs.

The functions of cytokines/chemokines in the Th2 immune network overlap considerably. The complex interactions in the molecular network may be related to the patients’ age, heredity, environment, and disease location. Ultimately the cytokine milieu at the barrier surfaces collectively contributes to the clinical observed symptoms and signs in various pathophysiological courses. Next, we will exclusively elaborate on the effects of TSLP in numerous immune-mediated skin conditions.

## TSLP and Cutaneous Immune-Mediated Disorders

TSLP has been shown to be a robust driver of Th2 responses at barrier surfaces. Accordingly, the involvements of TSLP in AD, a canonical type 2 cutaneous disorder, have been intensively studied. Besides, numerous investigations in both human and mouse skin implicated TSLP in a great variety of cutaneous disorders beyond AD. The following sections will describe the immune-mediated skin diseases linked with TSLP and the mechanisms of action through which TSLP produces its effect.

### AD

**Figure 2:**
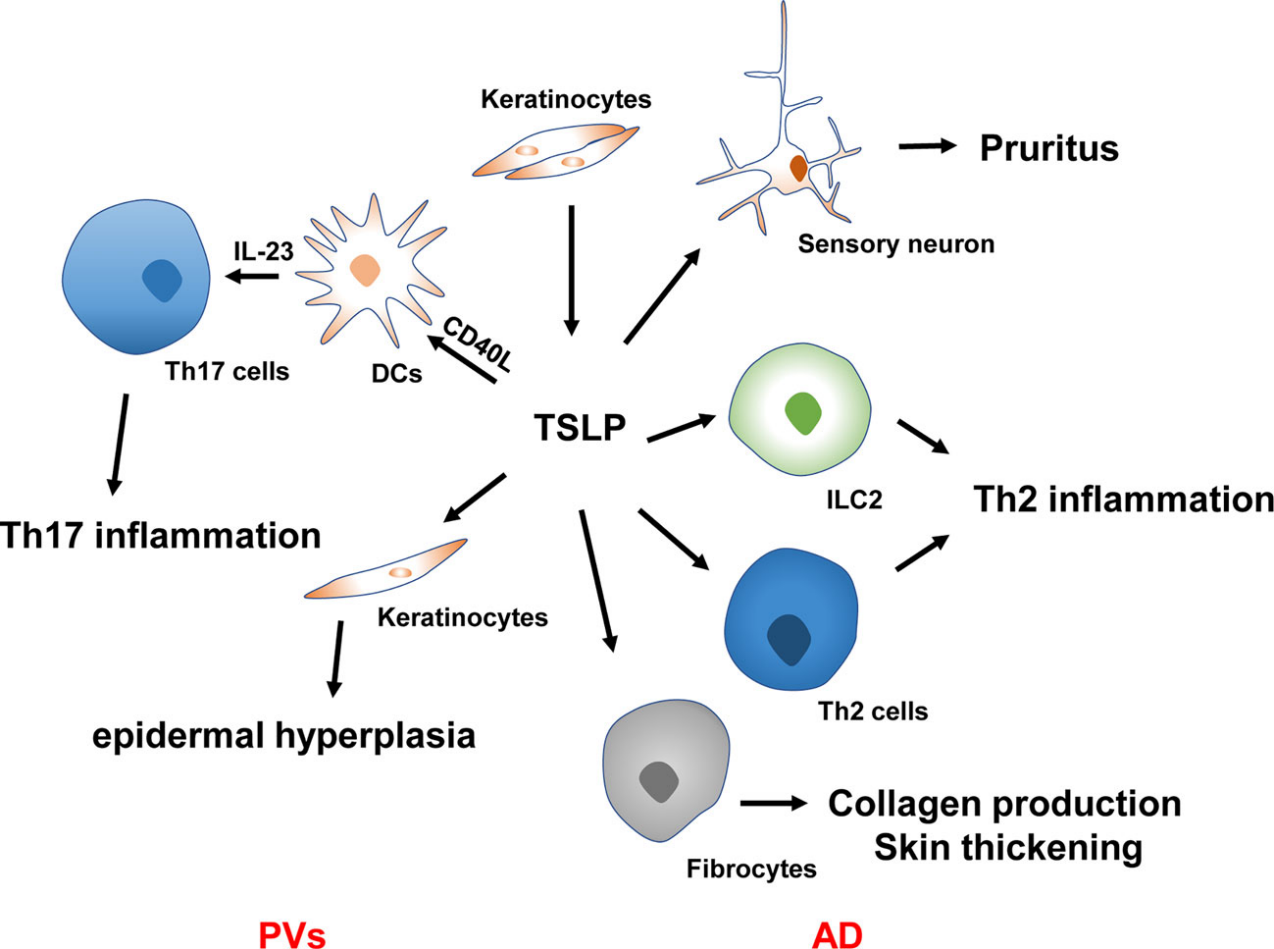
TSLP in the pathogenesis of AD and PVs. TSLP causes Th2 inflammation, pruritus, collagen production, and skin thickening in the pathogenesis of AD. TSLP synergy with CD40L to promote DC activation and IL-23 production, resulting in Th17 inflammation in the context of PVs. TSLP also regulates keratinocyte proliferation in the plaque of PVs, leading to epidermal hyperplasia. PVs, psoriasis vulgaris; AD, atopic dermatitis; ILC2, type 2 innate lymphoid cells; Th2 cells, type 2 T helper cells; Th17 cells, type 17 T helper cells; IL, interleukin.

AD, a relapsing inflammatory skin condition, is also called atopic eczema and features intense pruritus with a family history of atopies (49, 50). TSLP is closely linked with AD pathogenesis (**Figure 2**). Soumelis et al. first reported the involvement of TSLP in the immunopathogenesis of atopic diseases in 2002 (12). Through immunohistochemical analysis, the overexpression of TSLP in the keratinocytes of both acute and chronic lesions was identified in AD patients, while its expression was absent from non-diseased or non-lesional skin (12). TSLP expression in stratum corneum was more remarkable in AD patients than in controls. Furthermore, it was correlated with Scoring Atopic Dermatitis Index, especially with the stratum corneum hydration and dry skin score, indicating its overexpression corresponds to the epidermal barrier function (51). More importantly, keratinocytes-targeted TSLP overexpression induced AD-like lesions with an evident alteration in the infiltrate of Th2-related cells and an increased sera level of IgE (52). The persistence of AD is also related to the TSLP variation. Multiple studies have shown that specific gene polymorphism is closely associated with the genetic susceptibility of AD. A cohort study following more than 800 American children showed that the TSLPrs1898671 homozygosity contributed to the less-persistent AD in White children (53). More recently, another large-scale longitudinal cohort study found that the IL-7R variant rs11567725 can regulate the function of TSLP variants, suggestive of TSLP to be a potential genetic risk evaluation index of AD (54). These findings revealed that the epidermal production of TSLP is correlated with the clinically observed lesions, severity, and persistence of AD. TSLP-triggered DCs prime naïve T cells skewing Th2 immunity (12). Subsequent sensitization results in the occurrence of AD’s eczematous lesions.

Pruritus is considered a hallmark of AD and impairs a patient’s quality of life (QOL), leading to persistent scratching in AD patients (55). Physical damage attributed to chronic scratching significantly increased cutaneous TSLP levels (56). More importantly, TSLP may indirectly cause itching by inducing Th2-related cytokines that activate sensory neurons (57–59). TSLP was also demonstrated to function as a pruritogen. It acts directly on dorsal root ganglia neurons expressing TSLPR to trigger itch signals *via* the transient receptor potential cation channel A1 (60). A very recent research showed TSLP could induce the periostin secretion in keratinocytes that causes itching through the periostin receptor expressed on a subset of sensory neurons, indicating that TSLP may be involved earlier in AD development by inducing itching, scratching, and skin barrier dysfunction (61). Moreover, monoclonal antibodies targeting these cytokines have favorable functions in alleviating pruritus in AD patients (62, 63).

AD treatment has traditionally been confined to topical immunosuppressant use (50). Systemic immunosuppressants and phototherapy are applied in severe AD patients (50). Crisaborole ointment, a topical phosphodiesterase 4 inhibitor, and Dupilumab, a monoclonal antibody inhibiting IL-13 and IL-4 functions, have recently been approved by the US FDA for AD treatment (50, 64). Early-phase clinical trials targeting the immune pathway underlying the axis of TSLP-OX40L-OX40 have been tested in AD patients. A humanized antibody GBR830 targeting OX40, a costimulatory molecule expressed mainly on T cell subtypes, led to significantly lower clinical severity scores and decreased cutaneous AD gene signatures (Th1 signaling-IFN-γ and CXCL10, Th2 signaling-IL-31, CCL11, and CCL17, Th17/Th22 signaling-IL-23p19, IL-8, and S100A12) versus placebo (65). A small phase 2a clinical trial using class III topical corticosteroids plus tezepelumab, a TSLP monoclonal antibody that blocks the interaction of TSLP to its receptor complex, achieved a numerical but statistically non-significant reduction in eczema-severity scores over placebo at week 12, with more excellent week 16 responses (66). The clinical trials on TSLP suggest agents inhibiting TSLP and TSLP-related molecules may be helpful in AD patients.

### Psoriasis

Psoriasis vulgaris (PVs) is a chronic immune-related skin disorder substantially detrimental to patients’ QOL (67). Additionally, recent evidence demonstrated PVs as a systemic entity related to cardiovascular, endocrine, metabolic, gastrointestinal, renal, and consequentially psychological conditions such as depression and anxiety (68). Skin DC activation and IL-23 mediated induction of Th17 signaling is the primary pathophysiology of PVs (68, 69). TSLP’s role in PVs was initially elucidated in 2014 by Volpe and collaborators (70). Using immunohistochemistry, they revealed TSLP was significantly elevated in the untreated PVs patients’ epidermis (70). An *in vitro* study proved that TSLP and T cell-derived CD40L synergy contribute to DC maturation and subsequent IL-23 production by skin DCs and primary blood obtained from patients with PVs, which can be inhibited by a specific type 2 immune cytokine IL-4 in a dose-dependent manner (70). Several experiments further demonstrated that TSLP expression was more vigorous in skin lesions than in unaffected skin of PVs patients (71). Serum TSLP is also statistically increased and correlated with the severity of this disorder, suggesting its role as a prospective disease activity biomarker in psoriasis patients (71, 72). Although the experiments noted above propose that TSLP production is increased and plays a vital role in PVs development, another study showed minimal expression of TSLP in psoriatic lesions through immunohistochemical staining (73). The discrepancies may be attributed to the previous application of steroids suppressing TSLP production, thus pointing to the induction and function of TSLP are associated with PVs’ pathophysiology but not with therapeutical measures.

In addition to skin inflammation, PVs’ hallmarks include excessive keratinocyte proliferation, impaired differentiation, and epidermal hyperplasia in psoriatic plaque (68). Although various studies have highlighted the effects of TSLP in the skin DCs activation, its performances in the interaction of epidermal cells have rarely been investigated (11, 29). Recent studies have demonstrated TSLP, as an autocrine and paracrine factor, produced mainly by mutant bulge hair follicle stem cells and basal keratinocytes, can stimulate adjacent non-mutant epidermal cells to hyper-proliferate and express vascular endothelial growth factor α, thus contributing to skin inflammation and epidermal hyperplasia in PVs patients (74). Local injection of TSLP antibodies in the psoriasis-like mouse model led to skin inflammation regression, reduced epidermal hyperplasia, decreased vascular endothelial growth factor α expression, and epidermal inhibition of STAT-5 phosphorylation (74).

Psoriatic arthritis (PsA), a musculoskeletal inflammation associated with cutaneous manifestations, is a common comorbidity in PVs patients and develops in up to 40% of this disorder (68, 75). Angiogenesis occurs in the early stage of PsA, promoting the recruitment of leukocytes and ultimately destroying neighboring tissues (76, 77). *In vitro* studies using primary synovial fibroblasts (SFC) derived from PsA patients showed that TSLP was significantly elevated in PsA SFC-conditioned medium (76). Furthermore, fibroblasts from PsA synovium could induce the formation of endothelial cell tubes and increase angiogenic function through upregulation of TSLP, consistent with the results observed from cervical cancer previously (76, 78, 79). These data may support the blockade of SFC-derived TSLP as an optional method to alleviating abnormal angiogenesis in patients with PsA. Overall, these results extend the role of TSLP in regulating Th17 immune responses, keratinocyte proliferation, and angiogenic function in different microenvironments, with implications for other autoimmune conditions such as rheumatoid arthritis. More importantly, inhibition of the TSLP/TSLPR pathway could be beneficial for psoriasis patients and warrants additional clinical investigations.

### Bullous Pemphigoid

Bullous pemphigoid (BP), a highly prevalent type of skin autoimmune subepidermal blistering disease that mainly affects the elderly, typically manifests as pruritic vesicles and bullae (80). The pathophysiology of BP is still unclear, but autoantibodies to structured proteins in the dermal-epidermal junction and Th2 immunity are likely involved in this disorder (81, 82). Multiple studies have identified a markedly increased concentration of TSLP in skin lesions, blister fluid, and sera of patients with BP, especially in the lower layer of the epidermis (83–86). Furthermore, the levels of TSLP in serum correlated with those in blisters (86). These findings imply that TSLP is involved in the immunopathogenesis of BP and could be released by keratinocytes in the epidermis of BP.

Mice models have played a vital role in elucidating the mechanisms of BP (83). One study using a BP-like mouse model with the deletion of the NC16A domain of BP180 (termed △NC16A mice) showed spontaneous skin inflammation accompanied by elevated skin TSLP level and itching. This study demonstrated that △NC16A primary keratinocytes produce significantly more TSLP when stimulated with TNF-α *in vitro*. More importantly, increased expression of TSLP can also be observed in both local skin-specific △NC16A mice and basal keratinocyte-specific △NC16A mice, suggesting that BP180 dysfunction may induce basal keratinocytes to produce TSLP. Nevertheless, how BP180 dysfunction in keratinocytes triggers the release of TSLP and the molecular pathways/interactions in this process require further clarification.

Persistent pruritus is the most bothersome symptom in BP patients and significantly affects patient’s QOL (80). Given the critical role of TSLP in inducing itching in pruritic skin disorders, TSLP may also be involved in BP itching (83, 87). In the skin of △NC16A mice, significantly increased TSLP expression correlates with the severity of itching, while IgE or histamine is not involved in the mechanism underlying severe itching (83). Moreover, blockade of TSLP activity reduced scratching in △NC16A mice (83). However, the enhanced TSLP expression did not correlate with the severity of itching in BP patients (85). Thus, the pathogenic contribution of TSLP may be limited in BP-related itching.

The downstream mechanisms of TSLP in BP have received little attention. Li et al. showed that the number of DC-specific intercellular adhesion molecule-3-grabbing non-integrin (DC-SIGN)-positive DCs in BP lesional skin was higher than that in healthy controls and correlated with more robust TSLP expression in the epidermis and blisters of BP patients (86). In comparison, Langerhans cells (LCs) in the epidermis of BP lesional skin were less likely to be associated with increased TSLP expression (86). Moreover, DC-SIGN-positive DCs, but not LCs, expressing langerin, were positive for TSLPR (86). These data suggest that keratinocytes-derived TSLP may directly activate DCs. However, no studies thus far have verified the downstream mechanisms of the interactions between TSLP and DCs in BP patients.

### Systemic Sclerosis

**Figure 3:**
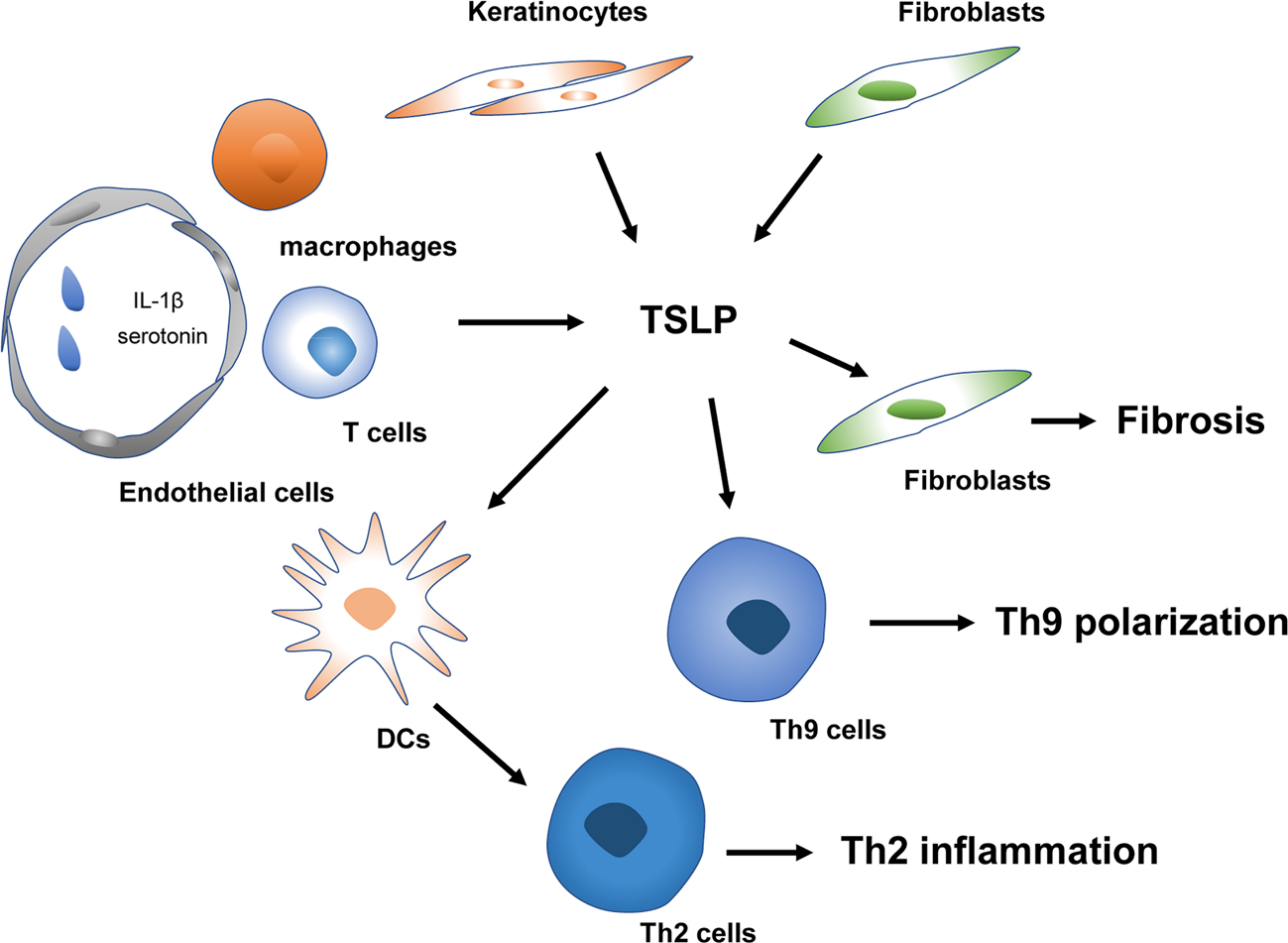
TSLP in the pathogenesis of SSc. TSLP mediates early Th2 and Th9 inflammation and late fibrosis of SSc. Platelets derived IL-1β and serotonin trigger TSLP released by vascular endothelial cells. TSLP contributes to Th2 and Th9 polarization in the early stage of SSc. Keratinocytes and fibroblasts releasing TSLP can modulate the fibrosis process in combination with TGF-β in the late phase of SSc. SSc, systemic sclerosis; IL, interleukin; Th2 cells, type 2 T helper cells; Th9 cells, type 9 T helper cells; DCs, Dendritic cells; TGF-β, transforming growth factor-β.

Systemic sclerosis (SSc), characterized by fibrosis of the skin and internal organs and vasculopathy, is an autoimmune disorder with high morbidity and mortality (88). It is generally believed vascular dysfunction, autoimmunity (especially the involvement of Th2 and Th17 responses), and fibrosis are the three essential characteristics of SSc (88–90). TSLP is also vital in the proinflammatory and profibrotic profiles of SSc (**Figure 3**). Multiple types of research have demonstrated expression of TSLP is significantly elevated in the skin of SSc-like mice models and patients (91–95). However, the contributors to the production of TSLP in these conditions remain controversial. Usategui et al. showed that TSLP overexpression was primarily linked to keratinocytes (92). In contrast, Christmann et al. failed to identify the difference between SSc and healthy donors (91). They demonstrated specific noticeable perivascular staining attributed to CD163+ macrophages and a few CD4+ and CD8+ T cells (91). The conflicting results may be ascribed to the specific study sources, and the disease phases since the infiltration of immune cells is commonly observed in the early stage of SSc (96). Furthermore, Truchetet et al. showed that TSLP expression is elevated in the epidermis and dermis of SSc patients. Interestingly, increased dermal numbers of TSLP-producing endothelial cells were strongly correlated with skin fibrosis (93). Also, serum TSLP was increased in SSc patients and associated with vascular dysfunction, indicating a possible earlier involvement of TSLP since vasculopathy occurs early in the progression of SSc (93).

Currently, the mechanisms underlying the triggers driving the expression of TSLP in SSc remain unknown. Toll-like receptors (TLR), IL-13, interferons, and TNF superfamily member LIGHT, have been demonstrated to induce TSLP release in SSc (96, 97). TLR activation, particularly TLR-3 triggering, induced TSLP expression by fibroblasts *in vitro* (92) and poly(I-C), a TLR-3 ligand, was confirmed to induce TSLP expression in infiltrating immune cells in a SSc murine model (91). Moreover, platelet-intrinsic IL-1β can contribute to skin fibrosis *via* the induction of TSLP production by human dermal microvascular ECs in a dose-dependent manner (93). LIGHT can directly induce TSLP in keratinocytes and synergize with transforming growth factor (TGF)-β to further affect TSLP expression (97). The specific potential of TSLP inducing TLR activation in SSc patients remains unknown. By exposing fibroblasts to plasma from SSc, Usategui et al. failed to detect enhanced TSLP expression (92). Further investigations are needed to clarify the precise mechanisms involved in the overexpression of TSLP in SSc.

The downstream effects of TSLP in SSc have not yet been fully elucidated. In the early phases of SSc, overexpression of TSLP was crucial for inducing profibrotic processes and proinflammatory molecules IL-13 and IL-17 (92). Furthermore, TSLP injection in the mouse skin induced clusters of genes assessed by microarray that IL-13 and TGF-β similarly enhance [e.g., plasminogen activator inhibitor 1 (PAI-1), thrombospondin 1, bone morphogenetic protein 1, and secreted phosphoprotein 1 (SPP-1)] (91). Moreover, TSLP deficiency inhibited TGF-β induced cutaneous fibrosis and abrogated several genes induced by TGF-β, involving PAI-1 and SPP-1 (91). *In vivo* and *in vitro* studies further demonstrated that TSLP strongly promoted Smad2 phosphorylation, a canonical TGF-β signaling pathway (91). These data suggest a complicated interaction between TGF-β and TSLP and the indispensable role of TSLP in TGF-β-mediated skin fibrosis. Detailed understanding of the interplay between the primary cytokines TGF-β, IL-13 and TSLP is crucial for future SSc treatment. Recent studies have demonstrated that TSLP and IL-4 increased dramatically in SSc and correlated immediately with an elevation of Th9 responses, which were also implicated in SSc by regulating autoimmunity and autoantibody production (94).

Besides promoting the early-phase development of SSc *via* increased autoimmune responses, TSLP has also contributed to fibrosis in the late phases of SSc. Truchetet et al. found that administration of TSLP in the dermis induces type I collagen synthesis and decreases the production of the collagenase matrix metalloproteinase 1, markedly changing their ratio, confirming a profibrotic activity for TSLP (93). The profibrotic profile in fibroblasts was consistent with the profibrotic gene signature previously identified in the mouse model by Christmann et al. (91). Moreover, TSLP-activated fibroblasts are capable of producing TGF-β, hence inducing TSLP, leading to a self-amplifying loop (96). Given the critical role of TSLP in the early phases (endothelial injury and autoimmune inflammation) and late phases (fibrosis) of SSc, therapeutically targeted modulation of TSLP may represent a novel antifibrotic strategy in SSc patients and warrants further investigation.

### Chronic Spontaneous Urticaria

Chronic spontaneous urticaria (CSU) is a chronic cutaneous disease that features recurrent itching wheals for more than six weeks without a definite eliciting factor (98). IL-25, IL-33, and TSLP affect mast cells, and notably, dermal TSLP-positive cells are markedly higher in the lesional skin of CSU than in non-lesional skin and healthy controls (99). These data suggest TSLP is implicated in the pathogenesis of CSU, and a blockade of TSLP/TSLPR signaling may provide an efficacious prospective treatment in CSU patients (100, 101).

### Behçet’s Disease

Behçet’s Disease (BD) is one of the most prevalent neutrophilic dermatoses characterized by recurring damage to the mouth, eyes, genitals, and skin (102). In severe cases, multiple systems and organs could be involved (102). The expression of TSLP and IL-33 mRNA increased dramatically in the skin lesions of BD and was correlated to the disease activity, indicating that the TSLP-IL-33 axis plays a crucial part in the pathogenesis of BD, linking the environmental triggers with systemic immune disturbance (103, 104).

### Vitiligo

Vitiligo, an acquired chronic depigmented skin disorder characterized by white macules’ development, features the loss of functional epidermal melanocytes (105). The autoimmune/autoinflammatory theory is the predominant hypothesis for vitiligo development since it is often associated with other autoimmune conditions such as thyroiditis (105, 106). TSLP polymorphisms may be essential genetic predispositions in vitiligo. An increased vitiligo susceptibility is linked with specific variants of TSLP identified by genetic studies (107, 108). In Korean patients, the TSLP-847C>T polymorphism was lower in vitiligo patients (107). The promoter activity of TSLP-847C decreased dramatically compared to TSLP-847T, which may enhance vitiligo susceptibility through decreased TSLP mRNA expression level, enhanced Th1 responses, and reduced Th2 activity (107). Further studies also provide robust evidence of a significant genetic association between vitiligo and TSLP (108). A case-control study was recently conducted to clarify the relationship between vitiligo and TSLP mRNA expression. A statistically lower TSLP mRNA expression in patients with generalized nonsegmental vitiligo than healthy controls was confirmed (109). Nonetheless, the expressions of TSLP at the protein levels in serum and lesional skin of patients with vitiligo have yet to be elucidated.

### Rosacea

Rosacea is a chronic inflammatory disorder that occurs in the middle of the face and mainly affects facial blood vessels and the sebaceous gland (110). TSLP expression was increased in sebaceous-rich healthy donors, but the DCs are in an immature state, suggestive of a tolerogenic activity of TSLP in sebaceous gland-abundant sites of healthy skin (111). In papulopustular rosacea, a loss of immune tolerance characterized by skin DC activation and alteration of inflammatory types accounts for the skin barrier abnormality and subsequent Th1/Th17 dominating immune responses (111–113). The sebum compositions and TSLP expression markedly decreased in the skin lesion of patients with rosacea, while IFN-γ and IL-17 elevated significantly. ﻿Dajnoki et al. hypothesized that the decreased sebum contents could potentially contribute to inhibiting tolerogenic TSLP production and changing the previous noninflammatory milieu, leading to the development of rosacea (111). However, there are few studies on the specific biological activity of TSLP in the pathogenesis of rosacea. Further investigations are needed to clarify the immunotolerant role of TSLP at the cutaneous barrier surfaces.

### Systemic Lupus Erythematous

Recently, pathway-based analyses have suggested that numerous potential pathways, including TSLP signaling, could be involved in the immunopathogenesis of systemic lupus erythematosus (SLE). SLE could also present as a mucocutaneous manifestation and can be only involved in the skin, as in cutaneous lupus erythematosus (114, 115). However, Truchetet et al. failed to determine elevated TSLP expression in either the skin or the circulating blood in patients with SLE (93). The mechanisms of the undiscovered factors that hinder TSLP expression in SLE have not yet been identified.

### Alopecia Areata

Alopecia areata (AA) is an autoimmune disorder with a lifetime incidence of nearly 2% (116). In addition to Th1-driven immune responses, the AA transcriptome showed an increased inflammatory profile of Th2 genes, including TSLP, indicating a possibility of Th2 signaling in AA patients (117). However, the TSLP-related pathways and Th2 immune responses were inhibited in a portion of severe AA patients (118). Topical treatment with diphenylcyclopropenone upregulated the TSLP-OX40L-IL13 axis in these individuals and promoted Th2-skewing immune responses, thus repairing equilibrium between Th1/Th2 ratios (118). A deep understanding of the precise pathogenic contribution of TSLP signaling in the pathogenesis of AA may yield novel targeted therapies for AA patients.

## Conclusion and Further Perspectives

TSLP is a critical regulator initially characterized by its ability to link innate immunity to type 2 acquired immune response at barrier surfaces. Emerging data show that TSLP can indirectly or directly affect a variety of non-immune and immune cells involved in a broad array of cutaneous immune-mediated disorders beyond just AD, vastly widening the function of TSLP in different skin conditions.

A group retrospectively reviewed the samples of eosinophil-rich dermatoses and identified that TSLP was diffusely expressed in arthropod assault, drug rash, AD, BP, and non-BP eosinophilic spongiosis (84). Based on these results and the findings from the literature, TSLP is closely involved in numerous skin conditions beyond just allergy and autoimmunity. TSLP expression is upregulated in patients with cutaneous T cell lymphoma (119). Tumor cells with TSLPR produce IL-13 and IL-4 in the presence of TSLP (119). Moreover, keratinocyte-specific deletion of the Notch pathway in mouse models of skin cancer showed a tumor-suppressive potential for TSLP (120, 121). Additionally, TSLP has also been implicated in keloid pathogenesis (122). These data add to the complicacy of TSLP immunomodulatory activities relying on the immune milieu. Understanding the pleiotropic properties of TSLP in various immune cascades will be essential in future research and development of effective TSLP-targeted therapies for these conditions.

## Author Contributions

The manuscript was written by S-HW with significant contributions from Y-GZ. All authors contributed to the article and approved the submitted version.

## Funding

This study was supported by the National Natural Science Foundation of China (grant number 81972944) and the Beijing Natural Science Foundation (grant number 7192166).

## Conflict of Interest

The authors declare that the research was conducted in the absence of any commercial or financial relationships that could be construed as a potential conflict of interest.
